# Anti-C1q antibodies antedate patent active glomerulonephritis in patients with systemic lupus erythematosus

**DOI:** 10.1186/ar2725

**Published:** 2009-06-10

**Authors:** Olivier C Meyer, Pascale Nicaise-Roland, Nolwenn Cadoudal, Sabine Grootenboer-Mignot, Elisabeth Palazzo, Gilles Hayem, Philippe Dieudé, Sylvie Chollet-Martin

**Affiliations:** 1 Rheumatology Unit, Bichat Hospital, APHP, 46 rue Henri Huchard, 75018 Paris, France; 2 Immunology Unit, Bichat Hospital, APHP, 46 rue Henri Huchard, 75018 Paris, France

## Abstract

**Introduction:**

Autoantibodies against C1q correlate with lupus nephritis. We compared titers of anti-C1q and anti-dsDNA in 70 systemic lupus erythematosus patients with (n = 15) or without (n = 55) subsequent biopsy-proven lupus nephritis.

**Methods:**

The 15 patients with subsequent lupus nephritis had anti-C1q assays during clinical flares (mean Systemic Lupus Erythematosus Disease Activity Index (SLEDAI), 10.0 ± 4.7; range, 3 to 22) before the diagnosis of lupus nephritis (median, 24 months; range 3 to 192). Among the 55 others, 33 patients had active lupus (mean SLEDAI, 10.3 ± 6.2; range, 4 to 30) without renal disease during follow-up (median 13 years; range 2 to 17 years) and 22 had inactive lupus (mean SLEDAI, 0; range, 0 to 3).

**Results:**

Anti-C1q titers were elevated in 15/15 (100%) patients who subsequently developed nephritis (class IV, n = 14; class V, n = 1) and in 15/33 (45%) patients without renal disease (*P *< 0.001). The median anti-C1q titer differed significantly between the groups (*P *= 0.003). Anti-C1q titers were persistently positive at the time of glomerulonephritis diagnosis in 70% (7/10) of patients, with no difference in titers compared with pre-nephritis values (median, 147 U/ml; interquartile range (IQR), 69 to 213 versus 116 U/ml; 50 to 284, respectively). Titers decreased after 6 months' treatment with immunosuppressive drugs and corticosteroids (median, 76 U/ml; IQR, 33 to 106) but remained above normal in 6/8 (75%) patients. Anti-dsDNA antibodies were increased in 14/15 (93.3%) patients with subsequent nephritis and 24/33 (72.7%) patients without nephritis (*P *= ns). Anti-C1q did not correlate with anti-dsDNA or the SLEDAI in either group.

**Conclusions:**

Anti-C1q elevation had 50% positive predictive value (15/30) and 100% (18/18) negative predictive value for subsequent class IV or V lupus nephritis.

## Introduction

Active proliferative glomerulonephritis is a serious manifestation of systemic lupus erythematosus (SLE) that may exist at disease onset or may develop later on during a flare. Clinical nephritis develops in about 50% of patients with SLE. Early diagnosis and rapid treatment of lupus nephritis are crucial to improving survival in SLE patients [1]. The prognostic significance of lupus nephritis indicates a need for identifying early biomarkers that predict nephritis development [2-4].

A major pathogenic hypothesis is that SLE involves defective renal clearance of immune complexes. Among immunological parameters, consumption of the early components of the classical complement pathway, such as C1q and C4, is strongly associated with the development of active SLE [5]. Low C1q levels, although occasionally caused by a rare genetic abnormality, are usually related to consumption by immune complexes such as dsDNA–anti-dsDNA or nucleosomes–antinucleosomes [6,7]. Another cause of low C1q levels is the presence of anti-C1q antibodies with the formation of C1q/anti-C1q immune complexes [8]. Anti-C1q antibodies have been described in patients with SLE [9-11] or other autoimmune diseases [12,13]. Their correlations with hypocomplementemia and glomerulonephritis suggest that anti-C1q may play a pathogenic role [14,15].

The aims of the present study were to determine the prevalence of anti-C1q antibodies in SLE patients with or without lupus nephritis after a long follow-up period and to test the predictive value of the anti-C1q assay for subsequent lupus nephritis. We also compared anti-C1q versus anti-dsDNA antibodies (another candidate for predicting lupus nephritis) regarding their ability to identify patients at high risk for lupus nephritis [2,16-19].

## Materials and methods

### Patients

In this single-center retrospective study, 70 adults meeting at least four of the 11 American College of Rheumatology criteria for the classification of SLE [20] were recruited. These 70 patients were chosen among 115 SLE patients followed longitudinally by one of the authors (OCM). Patients were selected based on the availability of stored serum samples.

In the 15 patients with lupus nephritis, we had stored (-20°C) serum samples obtained at least 3 months before the onset of clinical manifestations of nephritis, at a time when the disease was active (Systemic Lupus Erythematosus Disease Activity Index (SLEDAI) ≥ 4). Clinical nephritis was suspected if urinalysis showed proteinuria >0.5 g/dl on a 24-hour urine collection and/or hematuria or cellular casts with or without increased serum creatinine. Renal biopsies were performed for all 15 patients with clinical nephritis. The findings were classified according to the World Health Organization and International Society of Nephrology/Renal Pathology Society [21]. The renal disease was class IV in 14 patients and class V in one patient.

In the remaining 55 patients the disease was either active (SLEDAI ≥ 4) (n = 33) or inactive (n = 22) at the time of serum sampling, and there was no evidence of lupus nephritis (no low-grade proteinuria, hematuria, or cellular casts by routine periodic urinalysis) at any time during follow-up (mean, 11.3 ± 5 years; range, 2 to 17 years). The SLE activity at periodic serum sampling was defined based on the SLEDAI values in the medical records [22]. In patients with subsequent lupus nephritis, none of the renal parameters of the SLEDAI was present at the time of first serum sampling. These patients had active lupus without renal manifestations. At the time of the renal flare, the renal SLEDAI ranged from 4 to 16 points (one to three parameters). The main characteristics of the two patient groups are presented in Table 1.

**Table 1:** Characteristics of patients with systemic lupus erythematosus

	Active lupus with nephritis (n = 15)	Active lupus without nephritis (n = 33)	Inactive lupus without nephritis (n = 22)
Female/male	15/0 (100%)	28/5 (84.8%)	21/1 (95.5%)
Ethnicity			
Caucasian	13 (86.7%)	20 (60.6%)	19 (86.4%)
Black	2	9	3
Asian	0	4	0
Age at serum sampling (years)			
Median	30	28	35
Range	19 to 58	17 to 48	20 to 76
Duration of SLE (years) at serum sampling			
Median	6	2.1	10
Range	0.25 to 36	0.1 to 14.1	1.1 to 49
Duration of follow-up (years) (from SLE diagnosis to last visit)			
Median	13.5	13	19
Range	5.9 to 23	2 to 17	4 to 52
SLEDAI at first serum sampling (points)			
Median	10^a^	8	0
Range	4–22	4–30	0 to 3

SLE, systemic lupus erythematosus. ^a^Systemic Lupus Erythematosus Disease Activity Index (SLEDAI) was calculated before the onset of renal manifestations.

Patients with class IV or class III glomerulonephritis were treated with either intermittent intravenous cyclophosphamide infusions (0.6 g/m^2^/infusion) (13 patients) or oral mycophenolate mofetil (two patients) and with high-dose prednisolone (1 mg/kg/day) with or without initial intravenous methylprednisolone pulses (1 g). The single patient with class V glomerulonephritis also had central nervous system involvement, and was given intermittent intravenous cyclophosphamide infusions (12 infusions in all) after an incomplete response to azathioprine and high-dose oral corticosteroids.

In 11 of the 15 patients with nephritis, we obtained the anti-C1q and anti-dsDNA titers at the time of renal biopsy (n = 10) and/or after 6 months' immunosuppressive treatment (and: n = 7/ or: n = 1). Of the eight patients whose anti-C1q status was determined after immunosuppressive treatment, three patients were in complete remission and five patients were in partial remission of the nephritis. Partial remission was defined as any of the following: decrease in urinary protein excretion by >50% and to <3 g/day with a serum albumin level of ≥ 30 g/l, and either stable renal function or a serum creatinine decrease to <130 μmol/l for patients whose baseline serum creatinine was 130 to 260 μmol/l. (None of the patients had a baseline creatinine level >260 μmol/l.)

The hospital ethics committee approved the present study and all participants gave their written informed consent. The study was conducted in accordance with the recommendations of the Helsinki declaration and all investigations were those routinely required to evaluate the patients, each of whom gave informed consent to all procedures.

### Autoantibody assays

All autoantibody assays were performed on the stored serum samples during the same run, by technicians who were blinded to the patient groups.

IgG anti-C1q antibodies were determined using an ELISA with purified human C1q (Bühlmann Laboratories, Schönenbuch, Switzerland), according to the manufacturer's recommendations. Briefly, undigested purified human C1q served as the antigen, and sera were diluted and incubated in high-salt buffer (1 M NaCl). Optical densities were measured at 450 nm and converted into units (U/ml) by plotting against the autoantibody titer of the standards given by the manufacturer. The cutoff value for defining a positive test was determined by the manufacturer as 20 U/ml. With this cutoff value, 20% of 40 blood donors tested in our laboratory had positive tests. We calculated an optimized cutoff value at the 98th percentile (that is, 32 U/ml), which decreased the proportion of normal blood donors with positive tests to 6%, as reported in the literature [23].

IgG anti-dsDNA antibodies were determined using an ELISA with enzyme-linked immunoassay technology (PHADIA GmBH, Freiburg, Germany), according to the manufacturer's instructions. The cutoff value for a positive test result (15 U/ml), as determined by the manufacturer by testing samples from 400 healthy blood donors, corresponds to the 98th percentile. The fluorescent signal was converted to international units (IU/ml) by plotting the value against the autoantibody titer of World Health Organization standardized calibrators (Wo80) supplied by the manufacturer.

### Statistical analysis

Demographic, clinical, and histological characteristics are reported as the mean (standard deviation) or *n *(%), as appropriate. Results of the ELISAs are given as the median (interquartile range (IQR)). Nonparametric tests (Mann-Whitney *U *test, and Spearman's rank correlation test) and Fisher's exact test were used to compare the groups with and without lupus nephritis. Sigma Stat software, version 3.5 (Systat Software Inc., Chicago, IL, USA) was used for statistical analyses. *P *< 0.05 was considered significant.

## Results

### Study patients

The clinical characteristics of the study patients are presented in Table 1. No significant differences were found between the groups for the sex ratio, ethnicity, median age at serum sampling, median follow-up duration from SLE diagnosis to the last visit, or median SLEDAI at serum sampling in the two groups with active SLE. The only significant difference between the groups was a longer median duration of SLE in the nephritis group (6 years; range, 0.25 to 36 years) compared with the group without nephritis (2.1 years; range, 0.1 to 14.1 years) (*P *< 0.003). The median time from serum sampling to the diagnosis of lupus nephritis (15 patients) was 1.2 years (range, 0.3 to 16 years). The time from serum sampling to lupus nephritis was <12 months in 5 patients and ≥ 12 months in 10 patients (1 to 2 years in four patients, 2 to 5 years in five patients, and 16 years in one patient). Lupus treatment at the time of serum sampling in the 15 patients with subsequent nephritis involved low-dose prednisolone (<0.5 mg/kg/day) and hydroxychloroquine in eight patients, high-dose prednisolone (≥ 0.5 mg/kg/day) and intermittent intravenous cyclophosphamide in two patients, low-dose prednisolone and oral azathioprine (2 mg/kg/day) or methotrexate (0.4 mg/kg/week) in two patients each, and hydroxychloroquine with no corticosteroid in one patient.

### Anti-C1q antibodies

**Figure 1:**
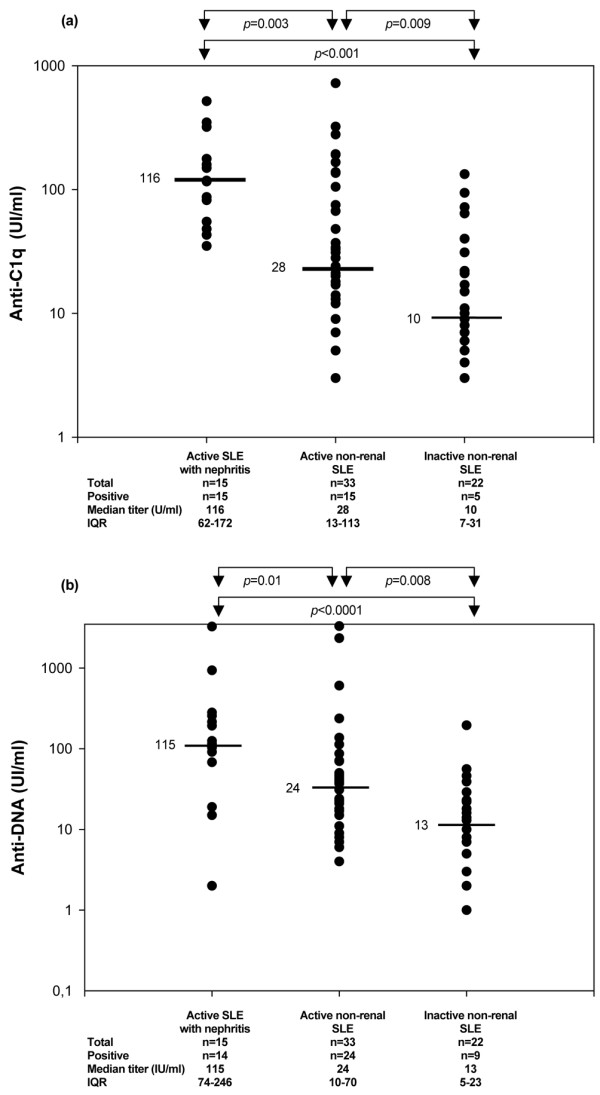
Anti-C1q and anti-dsDNA titers in systemic lupus erythematosus patients. Comparison of **(a) **anti-C1q and **(b) **anti-dsDNA titers in the three groups of systemic lupus erythematosus (SLE) patients. IQR, interquartile range.

Anti-C1q antibodies were found in all 15 (100%) patients with subsequent lupus nephritis, compared with 15 (45%) of 33 patients with active SLE but no nephritis (*P *< 0.001) and five (23%) of 22 patients with inactive SLE and no nephritis. The median plasma anti-C1q titer in the 33 patients with active non-nephritis lupus was 28 U/ml (IQR, 13 to 113 U/ml), compared with 116 U/ml (IQR, 612 to 172 U/ml) in the 15 patients with lupus nephritis (*P *= 0.003) and 10 U/ml (IQR, 7 to 32 U/ml) in the inactive non-nephritis group (Figure 1a). The sensitivity of anti-C1q antibody was 15/15 (100%) for subsequent severe lupus nephritis (class IV or class V). The specificity of the anti-C1q assay was 95.7%. The positive predictive value (PPV) for subsequent severe lupus nephritis was 15/30 (50%) and the negative predictive value (NPV) was 18/18 (100%).

The time between serum sampling and subsequent clinical lupus nephritis varied widely, from 3 months to 16 years. We therefore divided the nephritis patients into two subgroups, one with sampling-to-nephritis times <12 months (n = 5) and the other with longer times (n = 10). Anti-C1q antibody titers were not significantly different between these two groups (median, 82 U/ml vs. 117 U/ml).

**Figure 2:**
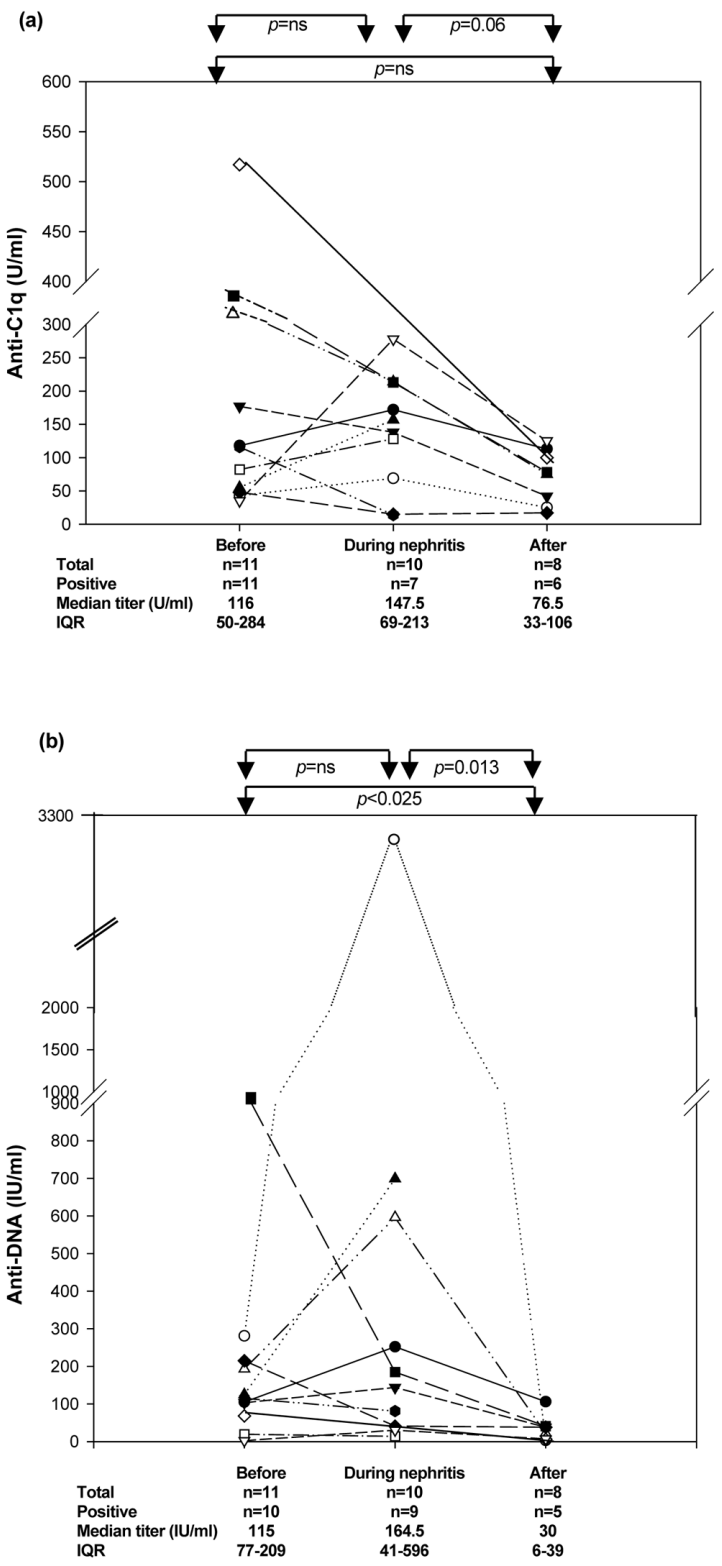
Follow-up of anti-C1q and anti-dsDNA titers in systemic lupus erythematosus patients with nephritis. Longitudinal follow-up of **(a) **anti-C1q and **(b) **anti-dsDNA titers in systemic lupus erythematosus patients with nephritis. IQR, interquartile range.

Serial anti-C1q assays were obtained in 11 of the 15 nephritis patients. At renal biopsy, anti-C1q antibodies were present with no significant titer variation in seven out of 10 patients; the antibodies were absent in two patients, one of whom was already treated with high-dose cyclophosphamide for transverse myelitis. After 6 months' immunosuppressive treatment, the anti-C1q titer decreased in eight out of eight patients (median, 76.5 U/ml; IQR, 33 to 106) (Figure 2a) but remained above normal in six out of eight patients.

### Anti-dsDNA antibodies

Anti-dsDNA antibodies were detected in 14 (93.3%) of 15 patients with subsequent lupus nephritis, compared with 24 (72.7%) of 33 patients with active SLE and no nephritis (*P *= ns) and nine (73%) patients with inactive SLE and no nephritis (*P *= ns). The median plasma anti-dsDNA titer in the 15 patients with nephritis was 115 IU/ml (IQR, 74 to 296 IU/ml), compared with 24 IU/ml (IQR, 10 to 70 IU/ml) in the 33 patients with active SLE and no nephritis (*P *= 0.01) and 13 IU/ml (IQR, 5 to 23 IU/ml) in the 22 patients with inactive SLE (*P *= 0.008) (Figure 1b). The NPV of anti-dsDNA antibodies for subsequent lupus nephritis was 9/10 (90%). The only patient who subsequently developed nephritis but had negative anti-dsDNA antibodies (2 IU/ml) had class V membranous glomerulonephritis 31 months after serum sampling.

At renal biopsy, the anti-dsDNA antibody test was positive (30 IU/ml). The sensitivity of anti-dsDNA for subsequent active proliferative lupus nephritis was 14/14 (100%), the PPV was 14/38 (37%), and the NPV was 9/10 (90%). Combining anti-C1q and anti-dsDNA led only to a slight improvement in the PPV (58.3% instead of 50%); both antibodies were positive in 10 out of 33 (30.3%) patients with active nonrenal lupus and in four out of 22 (18.2%) patients with inactive lupus. The NPV decreased to 95.8% when both antibodies were used in combination.

At the time of renal biopsy, nine out of 10 patients were positive for anti-dsDNA antibodies; the remaining patient, who previously had a weakly positive titer (19 IU/ml), had a negative titer at the time of biopsy (14 IU/ml) (Figure 2b). Finally, six out of eight patients had persistently low anti-dsDNA titers after 6 months of high-dose immunosuppressive drugs.

### Correlations

**Figure 3:**
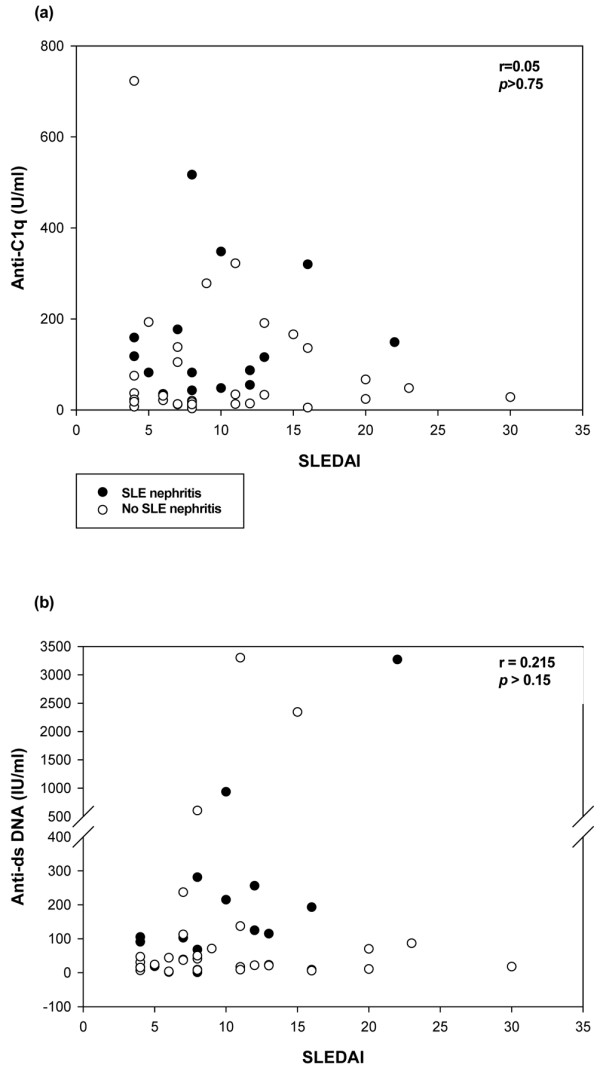
Correlation between the Systemic Lupus Erythematosus Disease Activity Index and anti-C1q and anti-dsDNA titers. Patients included those with active systemic lupus erythematosus (SLE) with (n = 15) and without (n = 33) subsequent lupus nephritis. The Systemic Lupus Erythematosus Disease Activity Index (SLEDAI) (before lupus nephritis) did not correlate **(a) **with anti-C1q antibodies in the overall population of 48 active SLE patients or in the 15 lupus nephritis patients, or **(b) **with anti-dsDNA titers in the overall population of 48 patients. In the 15 lupus nephritis patients, the SLEDAI showed a fair correlation with anti-dsDNA titers (*r *= 0.63, *P *< 0.02).

Anti-C1q titers did not correlate with anti-dsDNA titers in either group (data not shown). More specifically, anti-C1q titers in sera drawn before the onset of lupus nephritis did not correlate with anti-dsDNA titers (*P *= 0.6). In the two groups of patients with active SLE (with and without lupus nephritis, respectively), neither anti-C1q titers nor anti-dsDNA titers correlated with the SLEDAI value at serum sampling (Figure 3a,b). In the nephritis group, the anti-dsDNA titer correlated strongly with the SLEDAI value at sampling before the first renal manifestations (*r *= 0.63, *P *< 0.02). No correlation was found between anti-dsDNA titers and SLEDAI values in the 48 patients with active SLE (15 patients with and 33 patients without nephritis) (Figure 3b).

## Discussion

The aim of the present study was to determine whether anti-C1q antibodies predicted the subsequent development of lupus nephritis. Among previous studies, all but one [23] involved testing for anti-C1q at the time of the renal biopsy showing active proliferative or nonproliferative lupus nephritis [9,23-29]. In this setting, anti-dsDNA and other immunological parameters such as low complement hemolytic 50, C3, and C4 or the presence of antinucleosome showed moderate sensitivity and a moderate NPV for lupus nephritis [10,19,30-32]. We looked at anti-C1q titers at a time when the patients had no evidence of nephritis. Patients in whom nephritis developed subsequently were more likely to have anti-C1q antibodies (100%) than the other patients (45%), although the SLEDAI (excluding renal parameters) at the time of sampling was similar in the two groups before the first renal manifestation. The median anti-C1q titer was higher in the group with subsequent nephritis.

These data are consistent with the results of anti-C1q assays using a similar method in serum samples taken at the time of renal biopsy in 38 patients with proliferative (n = 26) or class V (n = 2) lupus nephritis [23]. Anti-C1q was found in 97% of patients with active proliferative nephritis, compared with 35% of lupus patients with inactive nephritis and 25% of lupus patients without nephritis [23]. Similar data were reported by Horak and colleagues in a series of 33 patients [33] and by Fang and colleagues in a series of 150 patients [29], all with lupus nephritis.

Our data indicate that the PPV of anti-C1q is only fair, as nearly one-half of the patients who still had no evidence of nephritis at follow-up completion had positive tests for anti-C1q, sometimes with very high titers. The mean follow-up was 11.3 years (median, 13 years) in this group, and we cannot rule out that nephritis will develop later on in some of the patients, particularly as the lower end of the follow-up range was only 2 years (three patients, including one patient with a very high anti-C1q titer).

With the caveat that we studied only a small number of patients, and that we studied only a single time measurement for a predictive value calculation of subsequent nephritis, the high NPV of anti-C1q is of interest. A high NPV is extremely helpful for identifying patients at low risk. All 14 patients with a subsequent diagnosis of class IV proliferative glomerulonephritis tested positive for anti-C1q at least 3 months before, and sometimes as long as 16 years before, the first clinical manifestation of renal disease. Anti-C1q antibody data at the time of renal biopsy showed good concordance with data obtained before the clinical manifestations of glomerulonephritis in seven out of 10 patients. One patient with an anti-C1q titer slightly above the cutoff value was diagnosed with class V glomerulonephritis 31 months later and had a high anti-C1q titer at the time of the renal biopsy. Another patient, whose anti-C1q titer was slightly above the cutoff value, was diagnosed 13 months later with class IV glomerulonephritis and had a negative anti-C1q test but was already treated with high-dose cyclophosphamide for extra-renal SLE manifestations.

The anti-C1q test may remain positive, however, even after 6 months of immunosuppressive therapy (five out of eight (62%) nephritis patients in our series, 30% in the study by Moroni and colleagues [32], and 47% in the study by Fang and colleagues [29]). In our study, this finding may be related to the fact that five out of eight patients achieved only partial responses. Other possibilities include inadequacy of the upper limit of normal determined in our laboratory in normal control individuals. We chose the 98th percentile as the upper limit of the normal, in keeping with many earlier studies. The high NPV of anti-C1q in our study is consistent with previous reports of anti-C1q values at the time of full-blown lupus nephritis [10,23,27-29,32-36] or before the diagnosis of lupus nephritis [23]. Moroni and colleagues reported a prospective 6-year study in 228 patients with lupus nephritis [32]. Among flares of proliferative lupus nephritis, 20% occurred at a time when anti-C1q titers were below the cutoff value, and 30% of patients were in remission of their nephritis at a time when anti-C1q titers were elevated. The best model for predicting renal flares was a combination of anti-C1q with C3 and C4 [32].

Anti-C1q titers did not correlate with the SLEDAI in patients with subsequent lupus nephritis. These antibodies should not be taken as a general marker of disease activity, in contrast to anti-dsDNA antibodies [37]. Our finding is in contrast with a recent report by Fang and colleagues that anti-C1q titers correlated well with the SLEDAI in 150 patients with renal SLE [29]. This discrepancy may be ascribable to differences in the patient populations and anti-C1q assays. In the study by Fang and colleagues, the IgG subclass distribution of anti-C1q antibodies suggested that IgG_2 _anti-C1q might be pathogenic and that IgG_3 _anti-C1q might be the most specific biomarker for monitoring disease activity (among patients in remission, 100% became negative for IgG_3 _anti-C1q compared with 53% negative for IgG_2 _anti-C1q).

## Conclusions

Our data suggest that anti-C1q may be a good serological marker for the subsequent development of active proliferative glomerulonephritis in patients with SLE. Patients without anti-C1q are at very low risk for severe proliferative glomerulonephritis (100% NPV in our study). Patients with anti-C1q antibodies have an approximately 50% risk for lupus nephritis within the next decade, and therefore require close renal monitoring.

## Abbreviations

dsDNA: double-stranded DNA; ELISA: enzyme-linked immunosorbent assay; IQR: interquartile range; NPV: negative predictive value; PPV: positive predictive value; SLE: systemic lupus erythematosus; SLEDAI: Systemic Lupus Erythematosus Disease Activity Index.

## Competing interests

The authors declare that they have no competing interests.

## Authors' contributions

OCM is the principal investigator, designed the investigation, collected the data, performed statistical analyses, and drafted the manuscript. PN-R collected the data, performed statistical analyses, and contributed to preparing the manuscript. NC and SG-M performed the ELISAs. EP, GH and PD helped to collect the clinical data. SC-M contributed to the preparation of the manuscript. All authors read and approved the final manuscript.

## References

[B1] Houssiau FA, Vasconcelos C, D'Cruz D, Sebastiani GD, de Ramon Garrido E, Danieli MG, Abramovicz D, Blockmans D, Mathieu A, Direskeneli H, Galeazzi M, Gul A, Levy Y, Petera P, Popovic R, Petrovic R, Sinico RA, Cattaneo R, Font J, Depresseux G, Cosyns JP, Cervera R (2004). Early response to immunosuppressive therapy predicts good renal outcome in lupus nephritis: lessons from long-term followup of patients in the Euro-Lupus Nephritis Trial. Arthritis Rheum.

[B2] Houssiau FA, D'Cruz D, Vianna J, Hughes GR (1991). Lupus nephritis: the significance of serological tests at the time of biopsy. Clin Exp Rheumatol.

[B3] Davis P, Cumming RH, Verrier-Jones J (1977). Relationship between anti-DNA antibodies complement consumption and circulating immune complexes in systemic lupus erythematosus. Clin Exp Immunol.

[B4] Lloyd W, Schur PH (1981). Immune complexes, complement, and anti-DNA in exacerbations of systemic lupus erythematosus (SLE). Medicine (Baltimore).

[B5] Walport MJ (2002). Complement and systemic lupus erythematosus. Arthritis Res.

[B6] Walport MJ (2001). Complement. Second of two parts. N Engl J Med.

[B7] Walport MJ (2001). Complement. First of two parts. N Engl J Med.

[B8] Sjoholm AG, Martensson U, Sturfelt G (1997). Serial analysis of autoantibody responses to the collagen-like region of Clq, collagen type II, and double stranded DNA in patients with systemic lupus erythematosus. J Rheumatol.

[B9] Mosca M, Chimenti D, Pratesi F, Baldini C, Anzilotti C, Bombardieri S, Migliorini P (2006). Prevalence and clinico-serological correlations of anti-alpha-enolase, anti-C1q, and anti-dsDNA antibodies in patients with systemic lupus erythematosus. J Rheumatol.

[B10] Siegert C, Daha M, Westedt ML, Voort E van der, Breedveld F (1991). IgG autoantibodies against C1q are correlated with nephritis, hypocomplementemia, and dsDNA antibodies in systemic lupus erythematosus. J Rheumatol.

[B11] Monova D, Monov S, Rosenova K, Argirova T (2002). Autoantibodies against C1q: view on association between systemic lupus erythematosus disease manifestation and C1q autoantibodies. Ann Rheum Dis.

[B12] Wener MH, Uwatoko S, Mannik M (1989). Antibodies to the collagen-like region of C1q in sera of patients with autoimmune rheumatic diseases. Arthritis Rheum.

[B13] Braun A, Sis J, Max R, Mueller K, Fiehn C, Zeier M, Andrassy K (2007). Anti-chromatin and anti-C1q antibodies in systemic lupus erythematosus compared to other systemic autoimmune diseases. Scand J Rheumatol.

[B14] Trouw LA, Groeneveld TW, Seelen MA, Duijs JM, Bajema IM, Prins FA, Kishore U, Salant DJ, Verbeek JS, van Kooten C, Daha MR (2004). Anti-C1q autoantibodies deposit in glomeruli but are only pathogenic in combination with glomerular C1q-containing immune complexes. J Clin Invest.

[B15] Flierman R, Daha MR (2007). Pathogenic role of anti-C1q autoantibodies in the development of lupus nephritis – a hypothesis. Mol Immunol.

[B16] Okamura M, Kanayama Y, Amastu K, Negoro N, Kohda S, Takeda T, Inoue T (1993). Significance of enzyme linked immunosorbent assay (ELISA) for antibodies to double stranded and single stranded DNA in patients with lupus nephritis: correlation with severity of renal histology. Ann Rheum Dis.

[B17] Steinman CR, Grishman E, Spiera H, Deesomochok U (1977). Binding of synthetic double-stranded DNA by serum from patients with systemic lupus erythematosus: correlation with renal histology. Am J Med.

[B18] Tron F, Bach JF (1977). Relationships between antibodies to native DNA and glomerulonephritis in systemic lupus erythematosus. Clin Exp Immunol.

[B19] Linnik MD, Hu JZ, Heilbrunn KR, Strand V, Hurley FL, Joh T (2005). Relationship between anti-double-stranded DNA antibodies and exacerbation of renal disease in patients with systemic lupus erythematosus. Arthritis Rheum.

[B20] Tan EM, Cohen AS, Fries JF, Masi AT, McShane DJ, Rothfield NF, Schaller JG, Talal N, Winchester RJ (1982). The 1982 revised criteria for the classification of systemic lupus erythematosus. Arthritis Rheum.

[B21] Weening JJ, D'Agati VD, Schwartz MM, Seshan SV, Alpers CE, Appel GB, Balow JE, Bruijn JA, Cook T, Ferrario F, Fogo AB, Ginzler EM, Hebert L, Hill G, Hill P, Jennette JC, Kong NC, Lesavre P, Lockshin M, Looi LM, Makino H, Moura LA, Nagata M (2004). The classification of glomerulonephritis in systemic lupus erythematosus revisited. Kidney Int.

[B22] Bombardier C, Gladman DD, Urowitz MB, Caron D, Chang CH (1992). Derivation of the SLEDAI. A disease activity index for lupus patients. The Committee on Prognosis Studies in SLE. Arthritis Rheum.

[B23] Trendelenburg M, Lopez-Trascasa M, Potlukova E, Moll S, Regenass S, Fremeaux-Bacchi V, Martinez-Ara J, Jancova E, Picazo ML, Honsova E, Tesar V, Sadallah S, Schifferli J (2006). High prevalence of anti-C1q antibodies in biopsy-proven active lupus nephritis. Nephrol Dial Transplant.

[B24] Grootscholten C, Dieker JW, McGrath FD, Roos A, Derksen RH, Vlag J van der, Daha MR, Berden JH (2007). A prospective study of anti-chromatin and anti-C1q autoantibodies in patients with proliferative lupus nephritis treated with cyclophosphamide pulses or azathioprine/methylprednisolone. Ann Rheum Dis.

[B25] Coremans IE, Spronk PE, Bootsma H, Daha MR, Voort EA van der, Kater L, Breedveld FC, Kallenberg CG (1995). Changes in antibodies to C1q predict renal relapses in systemic lupus erythematosus. Am J Kidney Dis.

[B26] Bigler C, Lopez-Trascasa M, Potlukova E, Moll S, Danner D, Schaller M, Trendelenburg M (2008). Antinucleosome antibodies as a marker of active proliferative lupus nephritis. Am J Kidney Dis.

[B27] Moroni G, Trendelenburg M, Del Papa N, Quaglini S, Raschi E, Panzeri P, Testoni C, Tincani A, Banfi G, Balestrieri G, Schifferli JA, Meroni PL, Ponticelli C (2001). Anti-C1q antibodies may help in diagnosing a renal flare in lupus nephritis. Am J Kidney Dis.

[B28] Marto N, Bertolaccini ML, Calabuig E, Hughes GR, Khamashta MA (2005). Anti-C1q antibodies in nephritis: correlation between titres and renal disease activity and positive predictive value in systemic lupus erythematosus. Ann Rheum Dis.

[B29] Fang QY, Yu F, Tan Y, Xu LX, Wu LH, Liu G, Shao FM, Zhao MH (2009). Anti-C1q antibodies and IgG subclass distribution in sera from Chinese patients with lupus nephritis. Nephrol Dial Transplant.

[B30] Cortes-Hernandez J, Ordi-Ros J, Labrador M, Bujan S, Balada E, Segarra A, Vilardell-Tarres M (2004). Antihistone and anti-double-stranded deoxyribonucleic acid antibodies are associated with renal disease in systemic lupus erythematosus. Am J Med.

[B31] Cervera R, Vinas O, Ramos-Casals M, Font J, Garcia-Carrasco M, Siso A, Ramirez F, Machuca Y, Vives J, Ingelmo M, Burlingame RW (2003). Anti-chromatin antibodies in systemic lupus erythematosus: a useful marker for lupus nephropathy. Ann Rheum Dis.

[B32] Moroni G, Radice A, Giammarresi G, Quaglini S, Gallelli B, Leoni A, Vecchi ML, Messa P, Sinico RA (2009). Are laboratory tests useful for monitoring the activity of lupus nephritis? A 6-year prospective study in a cohort of 228 patients with lupus nephritis. Ann Rheum Dis.

[B33] Horak P, Hermanova Z, Zadrazil J, Ciferska H, Ordeltova M, Kusa L, Zurek M, Tichy T (2006). C1q complement component and -antibodies reflect SLE activity and kidney involvement. Clin Rheumatol.

[B34] Horvath L, Czirjak L, Fekete B, Jakab L, Prohaszka Z, Cervenak L, Romics L, Singh M, Daha MR, Fust G (2001). Levels of antibodies against C1q and 60 kDa family of heat shock proteins in the sera of patients with various autoimmune diseases. Immunol Lett.

[B35] Fremeaux-Bacchi V, Noel LH, Schifferli JA (2002). No lupus nephritis in the absence of antiC1q autoantibodies?. Nephrol Dial Transplant.

[B36] Gunnarsson I, Ronnelid J, Huang YH, Rogberg S, Nilsson B, Lundberg I, Klareskog L (1997). Association between ongoing anti-C1q antibody production in peripheral blood and proliferative nephritis in patients with active systemic lupus erythematosus. Br J Rheumatol.

[B37] Kavanaugh AF, Solomon DH (2002). Guidelines for immunologic laboratory testing in the rheumatic diseases: anti-DNA antibody tests. Arthritis Rheum.

